# The effects of glucocorticoids and immunosuppressants on cancer outcomes in checkpoint inhibitor therapy

**DOI:** 10.3389/fonc.2022.928390

**Published:** 2022-08-23

**Authors:** Sebastian Bruera, Maria E. Suarez-Almazor

**Affiliations:** ^1^ Section of Immunology, Allergy and Rheumatology, Baylor College of Medicine, Houston, TX, United States; ^2^ Department of Health Services Research, The University of Texas MD Anderson Cancer Center, Houston, TX, United States

**Keywords:** checkpoint inhibitor therapy, glucocorticoids, immunosuppressants, cancer, immunotherapy

## Abstract

The emergence of checkpoint inhibitors has created a paradigm shift for the treatment of various malignancies. However, although these therapies are associated with improved survival rates, they also carry the risk of immune-related adverse events (irAEs). Moderate to severe irAEs are typically treated with glucocorticoids, sometimes with the addition of immunosuppressants as steroid-sparing therapy. However, it is unclear how glucocorticoids and immunosuppressants may impact cancer survival and the efficacy of immune checkpoint therapy on cancer. In this narrative review, we discuss the effects of glucocorticoids and immunosuppressants including methotrexate, hydroxychloroquine, azathioprine, mycophenolate mofetil, tumor-necrosis factor (TNF)-inhibitors, interleukin-6 inhibitors, interleukin-1 inhibitors, abatacept, rituximab, and Janus kinase inhibitors (JAKi) on cancer-specific outcomes in the setting of immune checkpoint inhibitor use.

## Introduction

Immune checkpoints are responsible for maintaining self-tolerance and preventing autoimmune disease in healthy individuals. However, in patients with cancer, tumor cells develop mechanisms that enhance these inhibitory pathways to evade an immune system response. Immune checkpoint inhibitors (ICIs) augment patients’ immune responses by inhibiting these checkpoint pathways. These agents have had tremendous success slowing tumor progression and increasing overall survival in different cancer types. However, as ICIs inhibit mechanisms responsible for self-tolerance, they may also cause inflammatory and immune-related adverse events (irAEs) that can affect virtually any organ system and can be life-threatening (1, 2). Although the pathogenesis of irAEs is still being understood, the activation of T-cells also increases the production of pro-inflammatory cytokines that are commonly seen in autoimmune diseases including interleukin (IL)-17, IL-21, and IL-6 (2).

The development of irAEs may indicate a greater immune response and could therefore be associated with improved tumor outcomes. Conversely, there is logical concern that treating irAEs with immunosuppressive agents may diminish the tumoral immune responses and negatively impact the efficacy of ICIs. This review summarizes the evidence on the use of different immunosuppressants for the treatment of irAE and their potential impact on overall survival and tumor responses in cancer patients receiving ICIs. As there is no data from clinical trials, our review is primarily based on observational studies and case series. It is important to note, that studies are difficult to interpret as irAEs have been shown to have better outcomes because of immune responses against tumors, however, irAEs may also potentially increase the risk of treatment-related morbidity and mortality, and their treatment with immunosuppressants could also impair tumor responses. In this review, we will discuss the effects of glucocorticoids, tumor necrosis factor (TNF)-inhibitors, and interleukin-6 inhibitors.

## Glucocorticoids

Glucocorticoids are generally prescribed as first line treatment for irAEs as recommended by professional guidelines the American Society of Clinical Oncology and European Society of Medical Oncology guidelines (3–5). These agents have strong immunosuppressant effects, and their potential biologic impact on cancer appears to be multifaceted. Furthermore, their effects may vary according to the type of malignancy (6).

We review the available evidence on the general tumoral effects of glucocorticoid therapy in patients with cancer receiving these agents, in those who are already on glucocorticoid therapy for other disorders when they start ICI therapy, and in those who receive glucocorticoids for the treatment of irAE.

Use of glucocorticoids at onset or early on after initiating ICI therapy

There have been multiple recent studies and systematic reviews that have explored the effects of glucocorticoid therapy in general, on tumor progression and overall survival. A summary of relevant studies is shown in **Table 1** (7, 10, 12, 16–18, 20, 21). These are mostly retrospective observational studies including patients who had been receiving an equivalent dose of prednisone of ≥10mg within the four weeks of initiation of ICIs for either irAEs, cancer-related symptoms, or other medical conditions such as chronic obstructive lung disease, autoimmune diseases, or radiation pneumonitis. These studies suggest that patients receiving glucocorticoids at baseline when receiving ICIs had decreased overall survival (OS) and progression-free survival (PFS) compared to those who did not receive glucocorticoids or received lower doses (9, 11, 17, 18). However, they did not adjust for reasons for baseline glucocorticoid use.

**Table 1:** Selected studies exploring the effects of glucocorticoids on cancer outcomes in the setting of immune checkpoint inhibitors.

Author	Year	Type of Study N: sample size	Cancer	Type of ICI	GC Start Date from ICI	GC Indication	Outcome	Summary of findings
Arbour 2018 (7) †	2011-2017	Retrospective N: 640	NSCLC	Single-agent PDL1	Within 30 days	Any	OS, PFS	GC associated with worse OS, PFS versus no GC. Adjustment for GC indication not performed.
Bruyère 2021 (8)	2007-2018	Retrospective N: 828	Any solid tumors	Anti PDL1 or CTLA-4	Any time	irAEs	OS, PFS	GC for irAEs associated with shorter PFS and OS – possibly mediated by interruption of ICI therapy.
Chasset 2015 (9)†	2010-2011	Retrospective N: 45	Melanoma	Ipilimumab	Within 30 days	Any	OS, ORS	GC at baseline associated with decreased OS. Analysis adjusting for GC indication not performed.
De Giglio 2020 (10)	2013-2018	Retrospective N: 413	NSCLC	All ICIs	Within 30 days	Any	OS, PFS	GC associated with worse OS if indication was for cancer symptoms but not for other reasons.
Drakaki 2020 (11)	2011-2018	Retrospective Claims N: 2213	NSCLC, Melanoma, Urothelial carcinoma	All ICIs	Within 30 days	Any	OS, TTNT	GC at baseline associated with worse OS in all three cancer types that persisted in multivariate models for NSCLC and urothelial cancer but not melanoma.
Fuca 2019 (12)†	2013-2017	Retrospective N: 151	NSCLC	All ICIs	Within 28 days	Any	OS, PFS	GC associated with decreased OS versus no GC. Analysis adjusting for GC indication not performed.
Li 2021 (13)	Search conducted November 2020	Systematic Review N=7155	NSCLC	Anti-PDL1	Pre-treatment with dexamethasone	Pre-treatment	OS, PFS	Pre-treatment with dexamethasone had improved PFS and OS. Pre-treatment was not associated with a lower rate of irAEs.
Maslov 2021 (14)	2014-2020	Retrospective N: 247	Any Metastatic Cancer	Anti-PD1/PDL1	Any time	Any	OS, PFS, ORR	GCs within two months of ICI was associated with worse OS and PFS than GCs after two months. Comparisons were not made with non-GC groups.
Paderi 2021 (15)	2016-2020	Retrospective N: 146	NSCLC, melanoma, renal carcinoma	Nivolumab, atezolizumab, pembrolizumab	Any time	Any	PFS	GCs for irAEs within 30 days was not associated with decreased PFS. GC for irAEs after 30 days was associated with improved OS.
Petrelli 2020 (16)†	Search conducted June 2019	Systematic Review N: 4045	Any cancers	All ICIs	Any time	Any	OS, PFS	GC associated with worse OS and PFS if indication was for supportive care or cancer symptoms, however, not for irAEs.
Riudavets (17)	2013-2018	Retrospective N: 267	NSCLC	Anti-PDL1 with possible Anti-CTLA4	Any time	Any	OS	GC associated with worse OS when used for cancer-related symptom but decreased irAEs. No decrease in OS when GC use for irAEs versus no GC.
Scott 2018 (18)†	2015-2017	Retrospective N: 210	NSCLC	Nivolumab	Within first 30 days of ICI.	Any	OS	GC associated with worse OS versus no GC. Analysis adjusting for GC indication not performed.
Skribek 2021 (19)	2016-2019	Retrospective N: 196	NSCLC	All ICIs	Any time	Any	OS	GC for cancer symptoms associated with worse OS but not when given for irAEs
Svaton 2020 (20)	N/A*	Retrospective N: 224	NSCLC	Nivolumab	N/A*	Any	ORS	Baseline GC associated with worse ORS compared to non-GC. GC Analysis adjusting for GC indication not performed.
Tokunaga 2019 (21)	N/A	Retrospective	Melanoma	Ipilimumab	Any time	Any	OS	GC started within 7 weeks of ipilimumab was associated with worse OS than GC started after 7 weeks, in patients with low tumor mutation burden.

ICI, Immuncheckpoint inhibitors; GC, Glucocorticoids; NSCLC, Non-small cell lung cancer; OS, Overall Survival; PFS, Progression Free Survival; irAEs, Immune-related adverse events; ORS, overall response rate; TTNT, Time to next treatment; N/A, Not Available.

^*^Data could not be extracted as only abstract was available.

^†^Overlap between in publications included within the systematic review.

Patients receiving glucocorticoids when initiating ICI may have increased tumor burden or comorbidities, which are associated with decreased survival. Therefore, some studies have examined if the reasons for glucocorticoid use at baseline can explain differences in OS and PFS, as opposed to attributing these effects to solely to the interaction between glucocorticoids and ICIs. Results have been mixed. For example, one retrospective study in patients with non-small cell lung cancer receiving ICIs compared 38 patients that received baseline steroids for cancer-related symptoms, 11 that received steroids for other indications (including irAEs or comorbidities), and 299 who were steroid naïve. Patients receiving glucocorticoids for cancer symptoms had worse outcomes than those receiving them for irAE (HR 4.53, 95% CI 1.8-11), clearly showing that the indication for therapy is an important confounder (12). These findings have also been replicated in a smaller retrospective study in Sweden with 196 patients with non-small cell lung cancer that showed early glucocorticoid use within four weeks of ICI for irAEs did not appear to affect the efficacy of ICIs or OS (19). This study did confirm previous findings that baseline use of glucocorticoids for cancer-related symptoms was associated with decreased OS. A recently published systematic review including twelve randomized clinical trials showed that the administration of dexamethasone with chemotherapy and ICIs as part of treatment protocols to mitigate adverse events had improved OS than treatment arms that did not include dexamethasone (13). However, these results of cannot be generalized to patients who receive chronic daily steroids as it only included pre-treatment doses with infusions.

There is scarce and conflicting evidence on the effects of concomitant therapy with glucocorticoids when starting ICI on the subsequent efficacy of these agents. Future studies need to be carefully designed as decreased survival may be related to pre-existing comorbidities and advanced tumor burden in those patients’ receiving glucocorticoids when initiating ICI. Furthermore, the current evidence is mostly confined to non-small cell lung cancer.

Use of glucocorticoids to treat irAEs

The associated effects of glucocorticoids on cancer outcomes when used for the treatment of irAEs is difficult to interpret. While glucocorticoids may mitigate the immune effects of ICIs, irAEs themselves may be a prognostic marker for enhanced tumor response to ICIs (15). Ideally, the comparison of outcomes between patients with the same irAEs treated with and without glucocorticoids in a clinical trial would provide more clarity but has obvious ethical considerations, as these agents are recommended as first-line treatment for irAE in clinical practice guidelines (3–5). For many of the available studies there are biases that are difficult to adjust for, especially in the setting of smaller sample sizes and rare events. A systematic review published in 2020 explored the association of glucocorticoids use and survival in patients with irAEs (16). This review included 16 studies with 4,045 patients. All studies but one was retrospective with a mostly low quality of evidence. This review showed that patients taking glucocorticoids had an increased risk of death (HR 1.54, 95% CI 1.24-1.91). However, after performing a subgroup analysis for the use of glucocorticoids for irAEs (9 studies with 926 patients), the HR decreased to 1.08 (95% CI 0.79-1.49) suggesting that the use of glucocorticoids for irAEs may not affect OS.

Other studies have investigated if the timing of glucocorticoids for irAEs may affect overall survival (within 30 days of ICIs versus after 30 days). One study of 156 patients examined OS in patients who received glucocorticoids within 30 days or after 30 days of initiation ICIs for irAEs. This study showed no association in glucocorticoid use and overall survival and a possible improved outcome for those who started steroids after 30 days – although this is prone to immortality bias (15). A separate retrospective study including 257 patients who received steroids for irAEs showed decreased overall survival if glucocorticoids were initiated within two months of ICI onset after adjusting for glucocorticoid indication including tumor site, brain metastases, and the type of irAEs (14).

Overall, the current data suggests that the use of glucocorticoids for irAEs may not have a large deleterious effect on overall survival. However, one needs to consider that most patients with irAE receive glucocorticoids initially and that the development of irAE may be associated with better response to ICI, as it may indicate a more robust immune response (15). Some data suggests that early use of glucocorticoids (within 60 days of ICIs) may be associated with worse cancer outcomes, but further studies are needed to validate this finding. Recent data also suggests that this may also be due in part due to interruption or discontinuation of ICIs while patients have irAEs (8). Finally, glucocorticoids are used at varying dosages and durations depending on the type and severity of the irAE being treated. Studies have been too small to account for these differences.

Glucocorticoids continue to be the preferred first-line agent or moderate to severe irAEs. However, their effect on tumor responses is not well-characterized as most studies are retrospective and cannot account for the interactions between irAEs and improved tumor outcomes versus glucocorticoids and hampering of ICI effects. The current evidence suggests that patients who are receiving glucocorticoids at the onset of ICI therapy may have decreased survival, but that this effect may also be confounded by indication bias, as patients on glucocorticoids are likely to have more advanced cancer and worse performance status – this needs to be validated in well-controlled prospective studies.

The evidence on the use of glucocorticoids for irAEs was not robust but overall, glucocorticoids did not appear to have a large deleterious effect on overall survival – an exception might be use early on, within 60 days of initiating ICI therapy. An additional issue confounding the effect of glucocorticoid use for irAE is that patients who develop immunotoxicity are likely to discontinue ICI more often, or earlier compared to those without irAE. Therefore, observed deleterious effects on survival could be related to early discontinuation of ICI therapy. As most studies have been retrospective, larger, prospective studies are needed to adjust for various confounders and to better establish potential differences according to dose and duration of glucocorticoid therapy during treatment with ICI.

## Conventional systemic disease modifying antirheumatic drugs and immunosuppressants

Glucocorticoids at the high doses that may be needed to treat irAEs can result in severe adverse events including infection, diabetes and cardiovascular disease among others. Also, because of the concern reviewed above on how their broad mechanism of action may impair the efficacy of ICI, there is a need for the use of steroid-sparing agents to be introduced early in the treatment of irAEs.

The use of conventional synthetic disease modifying antirheumatic drugs (csDMARDs) such as methotrexate or hydroxychloroquine has largely been confined to irAE arthritis and cutaneous disease. The csDMARDs have heterogenous mechanisms of actions that make it difficult to predict their potential interactions with ICI. In this section, we will discuss the use and of several csDMARD including methotrexate, hydroxychloroquine, azathioprine, and mycophenolate mofetil for the treatment of irAEs and their potential effect on ICI efficacy and tumor response.

In general, csDMARDs have not been associated with an increased risk for malignancy when used for the treatment of autoimmune diseases such as rheumatoid arthritis. An exception might be mofetil mycophenolate for which an association with lymphoma has been described in rare cases (22, 23).

Methotrexate is an immunomodulator that is frequently used in low doses for the treatment of inflammatory arthritis. Its proposed mechanism of action in rheumatoid arthritis is likely driven by adenosine signaling promoting an overall anti-inflammatory state. At low doses it is unlikely to cause critical immunosuppression. It has therefore been used widely in the treatment of irAE-arthritis (from CTLA-4, PD-1, or PD-L1 inhibitors alone or in combination) in patients in whom from glucocorticoids cannot be tapered successfully (24). Immune-related arthritis (from CTLA-4, PD-1, or PD-L1 inhibitors) responds well to methotrexate and does not seem to increase cancer progression (25, 26). It is not currently known whether continuing treatment with methotrexate in patients with rheumatoid arthritis affects the outcomes of patients with concomitant cancer receiving ICI.

Hydroxychloroquine is an immunomodulator that is frequently used in the treatment of systemic lupus erythematosus. Hydroxychloroquine impairs the fusion of autophagosomes with lysosomes (autophagy) and has been shown to decrease inflammation and improve outcomes in lupus (27). Interestingly, this autophagy effect is currently being investigated in multiple cancer types to see if it can sensitize cancer cells to chemotherapy (28). Hydroxychloroquine has also been used successfully in the treatment of IR-arthritis (from CTLA-4 inhibitor and/or a PD-1/PD-L1 inhibitor) (29). As hydroxychloroquine is not an immunosuppressant it is unlikely that it may affect tumor progression or the efficacy of ICI, however, the evidence is currently scarce and further research is needed.

Other immunosuppressants such as azathioprine and mycophenolate mofetil have been used for severe irAEs. The use of azathioprine is mostly restricted to irAE hepatitis when steroid-sparing agents are needed, as recommended by guidelines (3–5). Mycophenolate mofetil has also been used in the setting of irAE hepatitis, colitis and myocarditis. The efficacy of these agents is largely limited to case series and reports. It is unknown how these agents may affect tumor progression in the setting of ICIs.

While csDMARDs and synthetic immunosuppressants might be useful in the management of irAE without compromising tumoral immunity, a major drawback is the delay in the onset of response which can take weeks or months. For this reason, there is interest in other therapies, such as biologic agents that may have a faster onset of action.

## Biologic immunosuppressants

Several biologic agents targeting specific immune pathways and cytokines have been used in the treatment of irAE, most commonly tumor necrosis factor inhibitors (TNFi) and interleukin 6 inhibitors (IL6i).

### Tumor necrosis factor inhibitors

TNFi have been widely used for the management of various irAEs (from CTLA-4, PD-1, and/or PD-L1 inhibitors) such as colitis, myositis and inflammatory arthritis (30).

The role of TNF in the pathogenesis of malignancy is mixed, with both pro-tumor and anti-tumor effects. The ability of TNF to invoke apoptosis in tumor cells is well-established. This led to a trial in the 1980s examined administering TNF directly to invoke tumor apoptosis (31). However, this induced severe systemic toxicity. Separate studies then demonstrated paradoxical results showing that increased levels of TNF may also predispose to the development of malignancies (32–34).

While there were initial reports suggesting that therapy with TNFi increased the risk of developing cancer, especially lymphoma, in patients with rheumatoid arthritis and other autoimmune diseases. However, recent studies have not confirmed this association (34, 35). Conceivably, patients with more severe autoimmune disease are more likely to receive these drugs, and high inflammatory states have been associated with increased risk of cancer, possibly confounding the earlier reported associations. There is scarce data on the use of TNFi for the treatment of autoimmune disease in patients with concomitant cancer. Most studies have not shown worse survival outcomes with this treatment, but in most cases TNFi were given to patients with a history of malignancy, or who had been several years in remission rather than to those with active cancer undergoing treatment (36, 37).

### TNFi for the treatment of cancer

Because of the potential tumorigenic effects of TNF, a few trials have investigated the use of infliximab, a TNFi, for the treatment of advanced cancer (38, 39). While no major adverse outcomes were observed, there have not been other published studies reporting significant clinical benefits in the treatment of cancer.

### TNFi in patients receiving ICI

Preclinical studies of TNF and TNFi show varying effects on cancer cells and cancer immunity, which adds to the complexity of how these agents may impact the efficacy of ICI when used to treat irAE (40). *In vitro* studies have suggested that the addition of TNFi may augment the response against tumors when combined with immunotherapy (41). This led to a phase 1 trial that evaluated the use of TNFi in combination with ICIs (CTLA-4 and PD-1 inhibitors combination) for the treatment of melanoma (42). This study showed a high overall response rate with certolizumab, a TNFi, in combination with ICIs with a good safety profile. It is currently not known whether the use of TNFi during ICI may lower the risk of irAEs, although pre-clinical data suggest it may (43).

Two separate studies have shown mixed results on the effects of TNFi on cancer outcomes in melanoma, when used to treat irAEs. The Dutch Melanoma Treatment Registry included 1,250 patients of which 65 received TNFi for irAEs related to PD-1 and/or CTLA-4 inhibitors (44). This study showed that patients who received TNFi for the treatment for steroid-refractory toxicity had increased mortality compared to those who received steroids only (HR 1.61, 95% CI 1.03-1.51). However, this study did not adjust for specific irAEs (for example, patients receiving TNFi may be more likely to have colitis). A separate retrospective study of 27 melanoma patients who received infliximab for the treatment of immune-related colitis (with PDl-1 and/or CTLA-4 inhibitors) showed that cancer outcomes were not affected (45). An additional retrospective study of 327 patients with different malignancies that received ICIs included 35 patients receiving TNFi and glucocorticoids for colitis versus 44 that received glucocorticoids only. This study showed that those with colitis had improved OS compared to those without colitis regardless of whether treatment was with glucocorticoids only or with infliximab (46).

### Interleukin-6 Inhibitors

Interleukin-6 (IL-6), an acute phase reactant, is a deleterious prognostic marker for melanoma, as increased levels are associated with decreased survival in patients with this disease (47, 48). Increased levels of IL-6 are observed in patients with cancer or autoimmune diseases, and also in cancer patients who develop immune toxicity from immunotherapy, chimeric antigen receptor-T (CAR-T) therapy, or ICI. Therefore, there has been an increased interest in the use of IL-6 inhibitors, such as tocilizumab or sarilumab, to treat irAEs, with the expectation that they will be efficacious in the treatment of irAEs, without any deleterious effects on cancer outcomes. IL-6 inhibitors have been extensively used in patients with rheumatoid arthritis, and there is no evidence that they increase the risk of developing malignancy (34, 35).

One case series of 22 patients treated with tocilizumab (two of whom started treatment prior to receiving ICI) demonstrated a good safety profile and efficacy for the treatment of irAEs in melanoma (related to PD-1, PD-L1, and/or CTLA-4 inhibitors) (49). A separate case series of 34 patients with mostly lung cancer and severe irAEs (from a PD-1 inhibitor only) also showed good therapeutic potential and safety profile of tocilizumab (50). However, cancer outcomes were not examined. A recently published systematic review examined cancer outcomes after therapy with tocilizumab for irAEs (51). The review included 31 studies (20 articles and 11 abstracts) with a total of 91 patients who received tocilizumab for irAEs. Cancer outcomes were reported in less than 20% of the cases. While there is a limited number of patients reported, there have been no reports of disease progression after starting tocilizumab (51).

IL-6 inhibitors appear to be a potentially promising treatment for irAEs that may not affect the efficacy of ICI. However, the literature is scarce and more evidence from prospective studies is needed.

### Interleukin-1 inhibitors

Interleukin-1 (IL-1) is another cytokine that has been noted to be pro-inflammatory within the tumor milieu. The tumor microenvironment is typically pro-inflammatory, and inflammation is thought to instigate carcinogenesis and promote tumor growth and progression. Interleukin-1 potentially plays a key role in mediating these processes (52). A randomized controlled trial that examined the efficacy of canakinumab on atherosclerosis reported in an exploratory analysis that canakinumab potentially decreased the risk of lung cancer and improve lung cancer outcomes (53). The efficacy of anti-IL-1 therapy for cancer treatment is now being evaluated in various malignancies such as pancreatic, lung cancer, and melanoma among others (54). It is also being investigated as an adjunct therapy to ICI to potentially improve outcomes.

Interleukin-1 inhibitors are effective in the treatment of autoinflammatory diseases and gout, but are not used in other autoimmune diseases such as inflammatory arthritis or inflammatory bowel diseases. Some studies have shown that high levels of circulating IL-1 may be predictive of irAEs in melanoma (55). Therefore, there may be a role for IL-1 inhibitors in the treatment of irAEs as they are strong anti-inflammatory agents, and potentially could improve cancer outcomes. However, the current clinical data is scarce and more research is needed.

### Abatacept

Abatacept is a modified antibody that contains the extracellular CTLA-4 domain and prevents the activation of T-cells. Abatacept has been widely used in autoimmune diseases such as rheumatoid arthritis and psoriatic arthritis. The mechanism of abatacept is directly contradictory to anti-CTLA4 checkpoint inhibitors such as ipilimumab. Therefore, there is concern that the use of abatacept would strongly inhibit the tumor effects of checkpoint inhibitors and has not been widely used. Furthermore, studies of patients with rheumatoid arthritis receiving abatacept have shown an increase in the risk of developing malignancies, especially melanoma (35, 56).

The evidence of abatacept in the treatment of irAEs is limited to case reports in the setting of life-threatening disease with myocarditis and myasthenia gravis. These have shown that abatacept may lead to the successful treatment in these patients (the two cases referenced include nivolumab and nivolumab/ipilimumab) (57, 58). However, cancer outcomes have not been explored and there is no other observational data. Due to the theoretical deleterious effects of abatacept on tumor progression, its use should likely only be reserved for patients with severe life-threatening disease.

### Rituximab

Rituximab is a monoclonal antibody that targets CD20 to deplete B-cells. It used commonly in leukemia, lymphoma, and autoimmune disease (such as vasculitis and rheumatoid arthritis). There is no clear evidence that rituximab increases the risk of developing other malignancies when used for these diseases. Rituximab has been used in combination with chemotherapy and ICI in lymphoma with favorable safety profile but unclear tumor benefit in this setting (59).

The interaction between rituximab and ICI is unclear and there is a paucity on knowledge at this time as to how the depletion of B-cells may interact with the effects of ICI on inhibitory pathways – though preclinical evidence seems to suggest that B-cell depletion does not have an effect on tumor response to ICI (60).

A case report of a patient who received rituximab for the treatment of vasculitis with complete depletion of B-cells, after receiving a PD-1 inhibitor she had adequate tumor response and tolerability (61). Another retrospective of 10 who received rituximab for the treatment of cutaneous irAEs all had an excellent response, but cancer outcomes were not reported (62).

Although rituximab is recommended as therapy for selected ICI in practice guidelines, there evidence is confined to case reports and small case series, with no information on how it may impact ICI efficacy and cancer outcomes.

### Other biologic agents

There are other biologic agents targeting different cytokines (e.g IL-17, IL-23, IL-12 inhibitors) that are approved for use in patients with autoimmune disorders such as psoriasis, psoriatic arthritis, and ankylosing spondylitis. While their use for the treatment of irAE is of interest, as they target inflammatory cytokines, there is no evidence on their use other than in isolated case reports.

## Targeted synthetic therapies: Janus kinase inhibitors

The Janus tyrosine kinase (JAK) pathways are crucial for intracellular signaling in inflammatory responses. Dysregulation of the JAK axis is thought to play a role in autoimmunity and oncogenesis (63). Several JAK-inhibitors (tofacitinib, upadacitinib, baricitinib, and others) have been successfully used in autoimmune diseases such as rheumatoid arthritis and psoriatic arthritis. However, a recent randomized safety controlled trial comparing tofacitinib and adalimumab, a TNFi, in patients with rheumatoid arthritis, showed an increased risk for the development of malignancy in patients receiving tofacitinib, not meeting the pre-established non-inferiority criterion (64). There is no data evaluating the safety of JAKi in patients with autoimmune disease and concomitant cancer.

JAK signaling contributes to the pathophysiology of irAEs by establishing and perpetuating a pro-inflammatory environment. The use of JAKi for the treatment of irAE has been largely confined to case reports and case series. While there is interest in the use of these agents, there is also a concern that JAK pathways are instrumental to promote ICI anti-tumor responses, through their role in cytokine signaling, for instance interferon (65, 66). Therefore, there is a theoretical concern that the use of these inhibitors may decrease the efficacy of ICIs. Further research is needed to determine this relationship.

## Conclusion

In summary, the use of ICIs has created a paradigm shift in oncology and greatly improved cancer outcomes. However, their widespread use has also caused the emergency of irAEs. While irAEs may be associated with better oncologic outcomes due to enhanced immune activation, their treatment may impair immune tumoral responses. There is limited data on the potential tumor effects and safety of drugs used for the treatment of irAE. Glucocorticoids are the most recommended first-line agents but the data on their possible effects on cancer progression is conflicting, and confounded by tumor characteristics, comorbidities and dosage and duration of treatment which have not been adequately adjusted for in the available studies. The data for the most used steroid-sparing treatments (TNFi, and IL-6 inhibitors) is also scarce, but there have been no significant concerns on their use. However, large, prospective, well controlled studies, ideally randomized will be needed to determine the safety and efficacy of these agents for the treatment of irAE in patients with different tumors receiving ICI. Furthermore, more research is needed to determine if there may be any differences in the treatment of immunosuppression through different classes of ICIs (such as PD-1 and PD-L1 inhibitors versus CTLA-4 inhibitors).

## Author contributions

The authors confirm responsibility for the following: study conception and design, data collection, analysis and interpretation of results, and manuscript preparation. All authors contributed to the article and approved the submitted version.

## Conflict of interest

Author MS-A has received consultant fees from participation on advisory boards for Gilead, Avenue Therapeutics, ChemoCentryx. Current member of advisory board for Celgene. All activities unrelated to this work.

The remaining author declare that the research was conducted in the absence of any commercial or financial relationships that could be construed as a potential conflict of interest.

## Publisher’s note

All claims expressed in this article are solely those of the authors and do not necessarily represent those of their affiliated organizations, or those of the publisher, the editors and the reviewers. Any product that may be evaluated in this article, or claim that may be made by its manufacturer, is not guaranteed or endorsed by the publisher.
